# Supplementary material for: Differential targeting of the nucleosome surface and superhelical crevice sites with Ru and Os organometallic agents
Source: Nucleic Acids Res. 2026 Jul 25;54(14):gkag721. doi: 10.1093/nar/gkag721 (PMC13401049; doi:10.1093/nar/gkag721)
Supplement: gkag721_Supplemental_Files [file gkag721_supplemental_files.zip › Levy-etal_SI-050226.pdf]

SUPPLEMENTARY DATA

Differential Targeting of the Nucleosome Surface and Superhelical Crevice Sites with Ru and Os Organometallic Agents

Andrea Levy[1], Zenita Adhireksan[2,3], Thibaud von Erlach[1], Giulia Palermo[1,4], Alexey A. Nazarov[5,6], Christian G. Hartinger[7], Paul J. Dyson[5], Ursula Rothlisberger[1*] & Curtis A. Davey[2,3,8*]

[1] Laboratory of Computational Chemistry and Biochemistry, Ecole Polytechnique Fédérale de Lausanne, Lausanne CH-1015, Switzerland

[2] School of Biological Sciences, Nanyang Technological University, 60 Nanyang Drive, Singapore 637551

[3] NTU Institute of Structural Biology, Nanyang Technological University, 59 Nanyang Drive, Singapore 636921

[4] Present address: Department of Bioengineering and Department of Chemistry, University of California Riverside, 900 University Avenue, Riverside, CA, 52512, USA

[5] Institute of Chemical Sciences and Engineering, École Polytechnique Fédérale de Lausanne (EPFL), Lausanne, 1015 Switzerland

[6] Present address: Department of Chemistry, M.V. Lomonosov Moscow State University, Leninskie Gory 1/3, 119991 Moscow, Russia

[7] School of Chemical Sciences, University of Auckland, Private Bag 92019, Auckland 1142, New Zealand

[8] Małopolska Centre of Biotechnology, Jagiellonian University, 31-007 Kraków, Poland

[*] To whom correspondence should be addressed. Email: ursula.roethlisberger@epfl.ch (U.R.), curtis.davey@uj.edu.pl (C.A.D.)

CONTENTS: 1 Table & 5 Figures

Table S1. Binding energies for the arene and non-arene ligands in RuASN-C vs OsASN-C compounds obtained with different DFT functionals.

Binding energy (kcal/mol)	Compound	**BLYP**	**B3LYP**	**M06**	**M06L**
Arene	**Ru**ASN-C	-8.35	-11.28	-35.86	-36.38
	**Os**ASN-C	-25.85	-32.87	-56.64	-57.62
	**ΔΔE (Ru-Os)**	**17.50**	**21.59**	**20.77**	**21.24**
Non arene	**Ru**ASN-C	-30.62	-34.99	-44.94	-48.76
	**Os**ASN-C	-40.95	-46.73	-55.18	-59.41
	**ΔΔE (Ru-Os)**	**10.33**	**11.74**	**10.24**	**10.65**

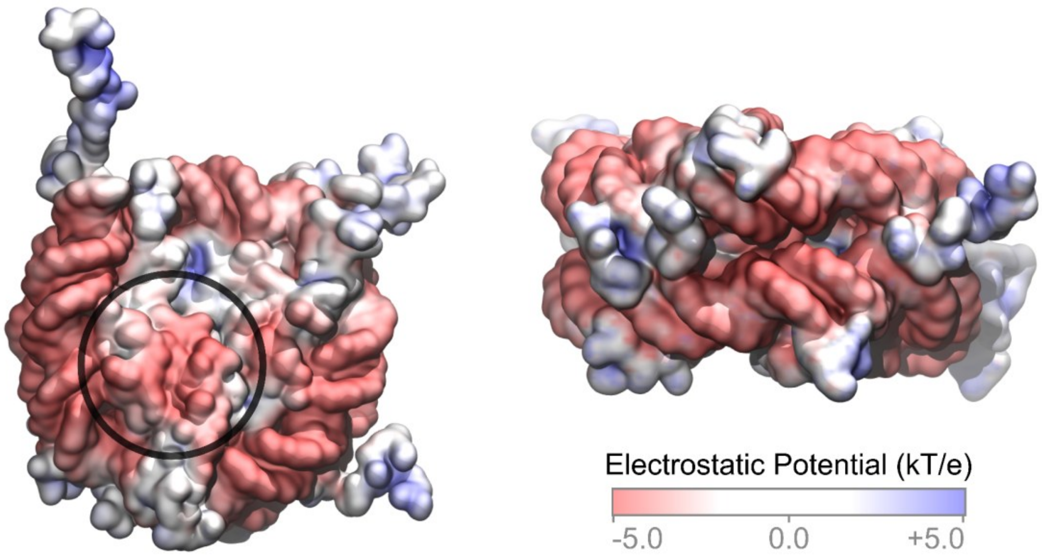

Figure S1. Electrostatic potential surface for the nucleosome core particle (NCP; PDB ID 1KX5; 39) computed with APBS VMD plugin, after protonation with H++ server (http://newbiophysics.cs.vt.edu/H++/). The acidic patch is highlighted with a black circle.

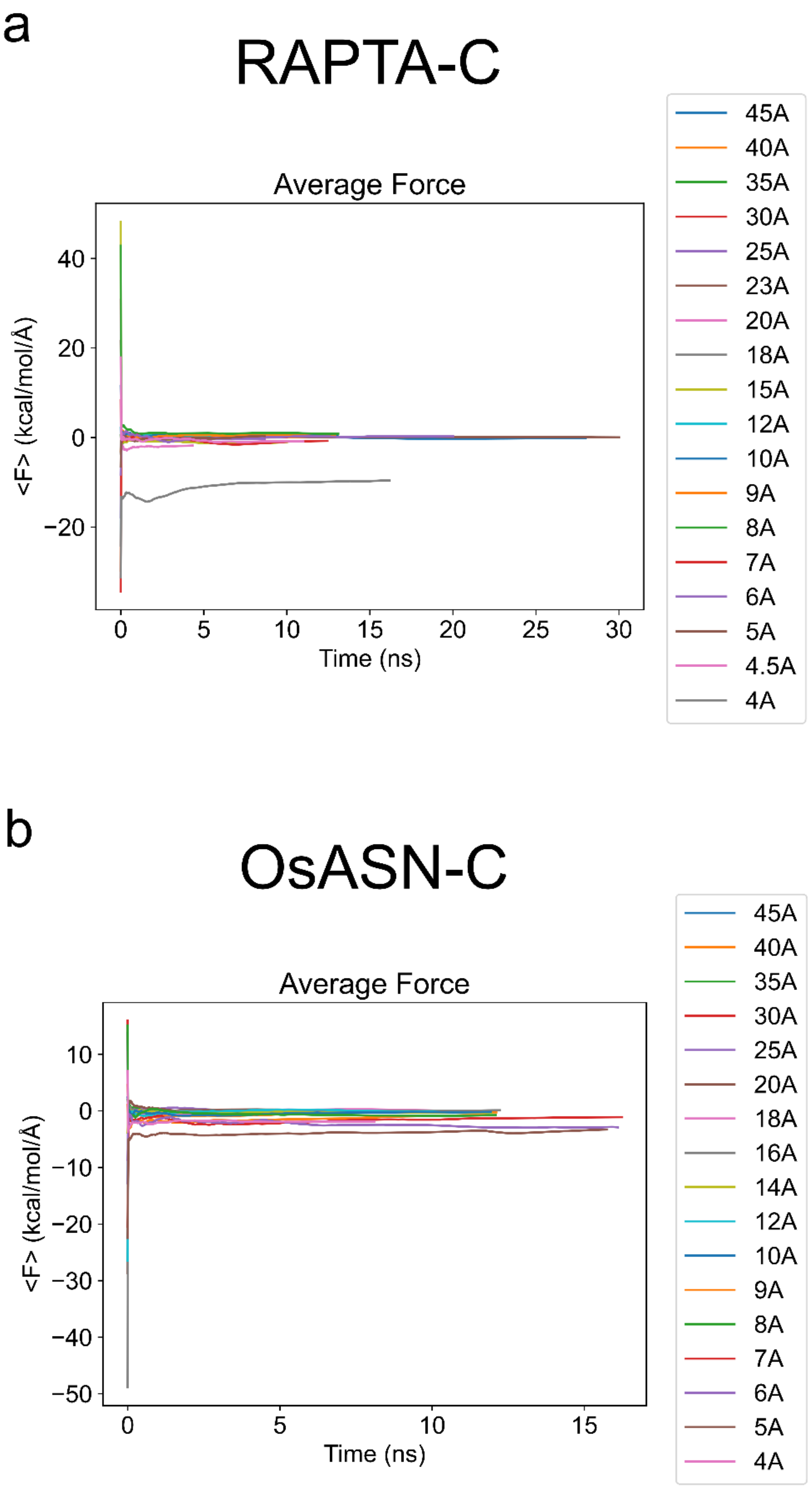

Figure S2. Convergence of the average force in the classical TI of the binding to the HIS site for RAPTA-C (**a**) and OsASN-C (**b**).

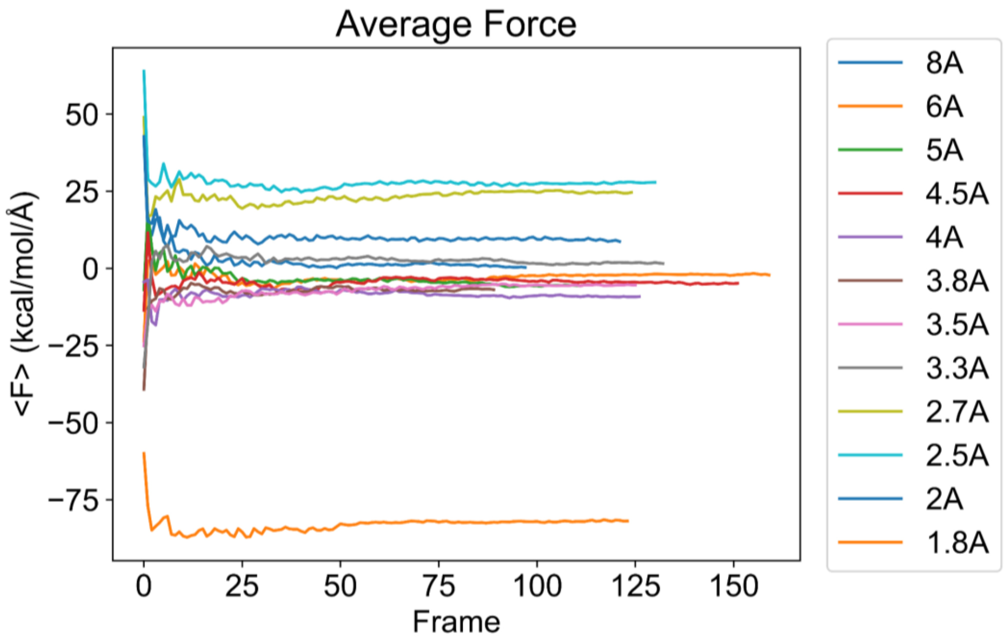

Figure S3. Convergence of the average force in the QM/MM TI of the binding to the HIS site for OsASN-C.

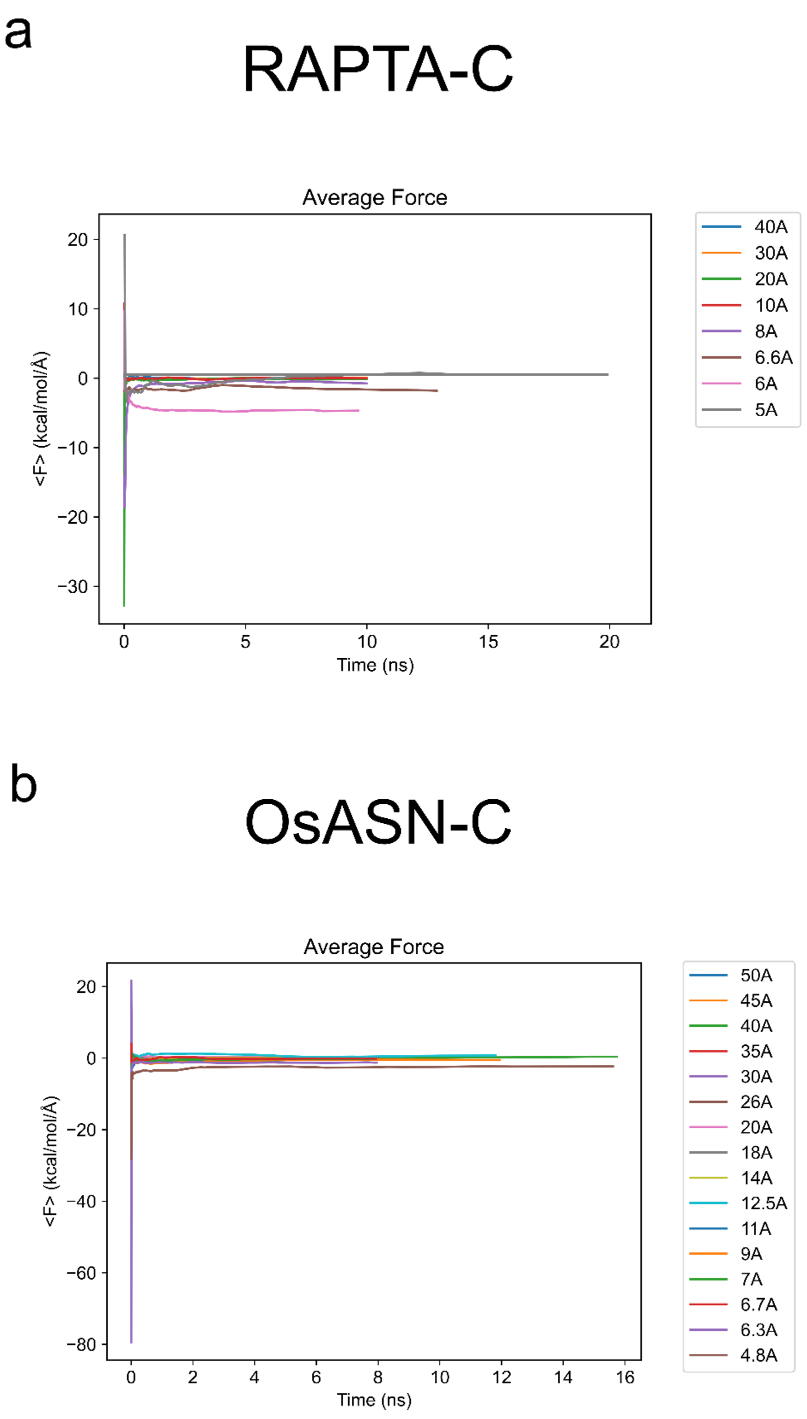

Figure S4. Convergence of the average force in the classical TI of the binding to the GLU site for RAPTA-C (a) and OsASN-C (b).

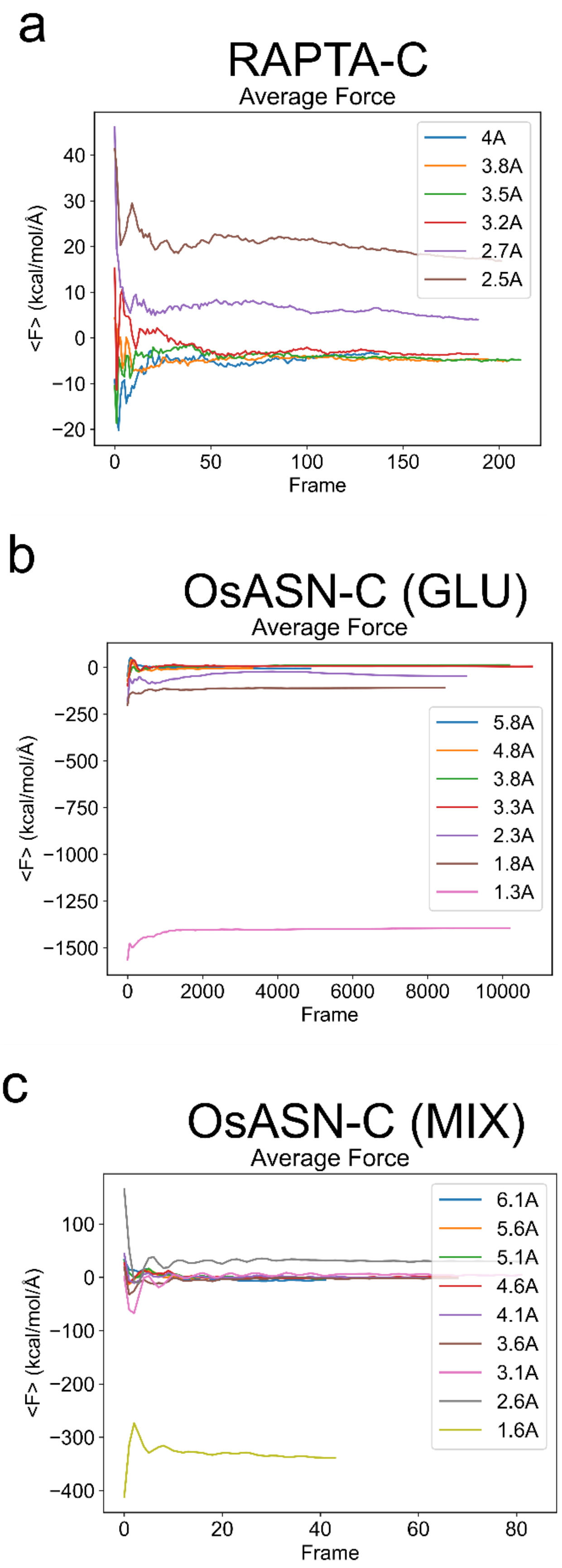

Figure S5. Convergence of the average force in the QM/MM TI of the binding for RAPTA-C to the GLU site (a), OsASN-C to the GLU site (b), and OsASN-C to the MIX site (c).
