# Supplementary material for: Differential targeting of the nucleosome surface and superhelical crevice sites with Ru and Os organometallic agents
Source: Nucleic Acids Res. 2026 Jul 25;54(14):gkag721. doi: 10.1093/nar/gkag721 (PMC13401049; doi:10.1093/nar/gkag721)
Supplement: gkag721_Supplemental_Files [file gkag721_supplemental_files.zip › Supplementary Video 1.docx]

Supplementary Video 1:

"Comparison of RAPTA-C and OsASN-C conformations between the bulk solvent and at the nucleosome superhelical crevice HIS site channel entrance. The two conformations have been extracted from TI windows at 40 Å (bulk solvent) and at 12 Å (channel entrance) and are displayed in alternating frames while the compound undergoes a 180° rotation to highlight structural differences and allow visualization from all perspectives."
